# Ciliary margin-derived BMP4 does not have a major role in ocular development

**DOI:** 10.1371/journal.pone.0197048

**Published:** 2018-05-08

**Authors:** Rebecca L. Rausch, Richard T. Libby, Amy E. Kiernan

**Affiliations:** 1 Neuroscience Graduate Program, University of Rochester Medical Center, Rochester, NY, United States of America; 2 Department of Ophthalmology, University of Rochester Medical Center, Rochester, NY, United States of America; 3 Center for Visual Sciences, University of Rochester, Rochester, NY, United States of America; 4 Department of Biomedical Genetics, University of Rochester Medical Center, Rochester, NY, United States of America; Massachusetts Eye & Ear Infirmary, Harvard Medical School, UNITED STATES

## Abstract

Heterozygous *Bmp4* mutations in humans and mice cause severe ocular anterior segment dysgenesis (ASD). Abnormalities include pupil displacement, corneal opacity, iridocorneal adhesions, and variable intraocular pressure, as well as some retinal and vascular defects. It is presently not known what source of BMP4 is responsible for these defects, as BMP4 is expressed in several developing ocular and surrounding tissues. In particular, BMP4 is expressed in the ciliary margins of the optic cup which give rise to anterior segment structures such as the ciliary body and iris, making it a good candidate for the required source of BMP4 for anterior segment development. Here, we test whether ciliary margin-derived BMP4 is required for ocular development using two different conditional knockout approaches. In addition, we compared the conditional deletion phenotypes with *Bmp4* heterozygous null mice. Morphological, molecular, and functional assays were performed on adult mutant mice, including histology, immunohistochemistry, *in vivo* imaging, and intraocular pressure measurements. Surprisingly, in contrast to *Bmp4* heterozygous mutants, our analyses revealed that the anterior and posterior segments of *Bmp4* conditional knockouts developed normally. These results indicate that ciliary margin-derived BMP4 does not have a major role in ocular development, although subtle alterations could not be ruled out. Furthermore, we demonstrated that the anterior and posterior phenotypes observed in *Bmp4* heterozygous animals showed a strong propensity to co-occur, suggesting a common, non-cell autonomous source for these defects.

## Introduction

Ocular development requires precise interactions between the neuroepithelium, surface ectoderm, mesoderm and neural crest. These cell types work in concert throughout embryonic and early postnatal development to form the multiple, specialized tissues of the eye [1,2]. Abnormalities in ocular tissue morphogenesis can lead to congenital diseases of both the anterior and posterior eye [3]. Anterior segment dysgenesis (ASD) is defined by ocular defects affecting the lens, cornea, iris, trabecular meshwork, ciliary body, and/or Schlemm’s canal [4]. In the posterior segment, abnormalities in retinal development often present as retinal coloboma or optic nerve hypoplasia [3]. Genetic studies have provided great insight into the identity of several molecules that control ocular development and dysgenesis [5], though the precise spatial and temporal function of many of these genes remains unclear. Furthermore, we have yet to fully understand how the multiple molecular networks act in concert to regulate specific inductive interactions between the diverse tissue types throughout eye development.

The Bone Morphogenic Protein (BMP) signaling pathway is involved in embryogenesis throughout the body (reviewed in [6]). In the eye, BMP signaling is required for development of the lens [7], ciliary body [8], and retina [9]. However, the specific spatiotemporal requirements of individual BMP ligands, receptors, and effector proteins are not yet fully understood. In humans, haploinsufficiency caused by heterozygous *Bmp4* mutations results in ASD and retinal anomalies, at times occurring within the same eye of a patient [10,11]. Similarly, mice heterozygous for a null allele of *Bmp4* have multiple phenotypes characteristic of ASD, such as irregular pupils, corneal haze, iridocorneal adhesions, and increased intraocular pressure [12]. Defects in the posterior segment are observed in these mice as well, including aberrant retinal vasculature, optic nerve abnormalities, and disrupted retinal lamination [12]. Thus, analysis of heterozygous mutations in both humans and mice indicate that BMP4 plays an important role in ocular development, though in which tissue type(s) and at what developmental time points BMP4 expression is required remains to be determined.

In the mouse, BMP4 is expressed early in the dorsal optic vesicle and subsequently in the developing central and peripheral optic cup, which gives rise to retinal neuroblasts and the aqueous humor-producing ciliary body, respectively [7,13,14]. Recently, a report showed that prior to optic cup formation, targeted deletion of *Bmp4* in the optic vesicle using *Rx*Cre, led to a marked decrease in retinal-specific genes, which were replaced by retinal pigmented epithelium markers [9]. Importantly however, the early *Bmp4* deletion also led to a failure of lens induction [9], consistent with previous reports demonstrating that lens induction requires optic vesicle-derived BMP4 around embryonic day (E)9.0 [7,15,16]. The *Rx*Cre used in the prior study has additionally been shown to be expressed in the lens, preventing a definitive conclusion as to which tissue is providing the required source of BMP4 [9,17,18]. Thus, because it has previously been shown that lens induction must proceed normally to establish proper neural retinal cell fates [19–21], it is unclear whether loss of neuroepithelial identity was caused directly by deletion of BMP4 in the optic vesicle or whether it was caused secondarily by failure of lens induction.

Following optic vesicle invagination and lens induction, BMP4 is expressed in the optic cup and in the ciliary margin, an embryonic tissue that gives rise to the ciliary body and iris [7,8,14,20,22]. As these structures are often affected in ASD, we were interested in determining whether BMP4 is critical for their development. Thus, to explore the role of BMP4 in the ciliary margin, we conditionally deleted *Bmp4* using two spatiotemporally targeted Cre recombinase mouse lines expressed in the optic cup. At the time of deletion, lens formation has already been induced [15], but ciliary epithelium and retinal cell fate determination have not yet occurred [23,24]. Thus, our mouse models directly tested the role of BMP4 in optic cup-derived tissues, in addition to possible non-cell-autonomous effects on neighboring structures. To our surprise, optic cup-derived BMP4 was found to be dispensable for the formation of all ocular tissues.

## Materials and methods

### Animals

All experiments were conducted in adherence with the Association for Research in Vision and Ophthalmology's statement on the use of animals in ophthalmic and vision research and were approved by the University of Rochester's University Committee on Animal Resources. Mice carrying a floxed allele of *Bmp4* [25], αCre [19], *Six3*Cre [26], *CMV*Cre [27], and *Rosa26-CAGTdtomato* [28] have been previously described. Peripheral optic cup neuroepithelium-specific conditional mutants were obtained by crossing animals carrying αCre*; Bmp4*^*fl/+*^ and *Bmp4*^*fl/fl*^ genotypes. Central optic cup neuroepithelium-specific conditional mutants were obtained by crossing animals carrying *Six3*Cre*; Bmp4*^*fl/+*^ and *Bmp4*^*fl/fl*^ genotypes. All conditional knockout animals were genotyped for the *Bmp4* recombined allele, and those with germline deletion were excluded from further analysis (S1 Fig). Crossing animals carrying a ubiquitous, non-inducible *CMV*Cre and *Bmp4*^*fl/fl*^ genotypes resulted in *Bmp4* germline heterozygous mutants. *α*Cre, *Six3*Cre, *CMV*Cre and *Bmp4*^*fl/fl*^ were each maintained on a C57BL/6J background (backcrossed ≥7 generations for αCre, ≥25 generations for *Six3*Cre, ≥25 generations for *CMV*Cre, and ≥3 generations for *Bmp4*^*fl/fl*^*)*. Cre-negative littermates were used as controls in each experiment. Established PCR protocols were used to genotype DNA samples extracted from the toes of P7 pups with allele-specific primer sets. Mice were housed in a 12-hr light/dark cycle and were fed chow and water ad libitum. Housing and handling of animals was performed in accordance with the Association for Research in Vision and Ophthalmology’s statement on the use of animals in ophthalmic research and approved by the Committee on Animal Resources at The University of Rochester Medical Center.

### Tissue processing and histology

Eyes were enucleated from adult mice and fixed in either 4% paraformaldehyde in 1XPBS for two hours (for subsequent cryo-preservation) or 2.5% paraformaldehyde; 2% glutaraldehyde in 1XPBS overnight (for subsequent plastic embedding). Prior to cryo-sectioning, the posterior segment was removed below the limbus and the lens was extracted. Eyes were submerged in 30% sucrose in 1XPBS for two days, embedded in tissue freezing medium, sectioned at 14μ (HM550 Microm Cryostat), and prepared for immunohistochemistry. Eyes to be sectioned in plastic were left intact after enucleation, dehydrated with a series of ethanol washes, embedded in Technovit 7100 hardener (Kulzer), sectioned at 2.5μ (HM 355S Automatic Microtome), and stained with hematoxylin and eosin (Multiple Stain Solution, Polysciences). Retinas taken for wholemounts were removed from the fixed eye and processed for immunohistochemistry.

### Immunohistochemistry

For sections, 14 μm cryo-sections were blocked for two hours with 10% horse serum in 0.1% Triton-X in 1XPBS followed by primary antibody staining with mouse anti-α-smooth muscle actin (Chemicon International; 1:100) overnight at 4° C. A secondary antibody conjugated with Alexa Fluor 488 (Donkey anti-Mouse Alexa 488, Thermo Fisher Scientific; 1:1000) was used the following day for two hours at room temperature. Three 1XPBS washes were performed between antibody incubations. Retinal wholemounts were blocked overnight with 10% horse serum in 0.1% Triton-X in 1XPBS followed by primary antibody staining with mouse anti-TUJ1 (Covance; 1:1000) for three nights at 4° C and incubation in Alexa Fluor 488 secondary antibody for two nights at 4° C. Following staining, wholemounts were cut allowing retinas to lie flat with the inner nuclear layer facing up. All fluorescent images were visualized and photographed on a Zeiss M1 Epifluorescent microscope with Axiovision software. Cell counts on retinal wholemounts were performed as described previously [29].

### *In situ* hybridization

Perinatal heads (P1) were fixed in 4% paraformaldehyde in 1XPBS for 24 hours, then submerged sequentially in 10%, 20%, and 30% sucrose, each for 24 hours at 4° C prior to embedding in tissue freezing medium. Heads were sectioned at 14μ and processed using digoxygenin (DIG)-labeled antisense probes (as described previously [30]) followed by enzymatic detection according to manufacturer’s protocols (Roche). The *Bmp4* plasmid was kindly provided by Dr. Rulang Jiang [31].

### *In vivo* imaging

Imaging of the anterior segment was performed on awake mice using a slit lamp biomicroscope (Topcon) equipped with digital camera (Nikon). Photographs of the retinal vasculature were obtained with a Micron III mouse retinal imaging system (Phoenix Research Labs). Mice were first anesthetized with an intraperitoneal injection of ketamine/xylazine mix (5μL/g) and then injected with fluorescein (25% Fluorescein Sodium; 0.2μL/g) immediately prior to fundus angiography.

### Intraocular pressure measurement

IOP measurements were obtained with a TonoLab tonometer. IOP was taken approximately 3 minutes after anesthesia administration (intraperitoneal injection of ketamine/xylazine mix (5μL/g)) as previously described [32].

### Optic nerve wholemount

Adult animals were euthanized and decapitated, and heads were submerged in pre-filled buffered 10% formalin containers (Fisherbrand) overnight. The next day, brains were dissected with optic nerves kept intact, and photographed under a dissection microscope using an iPhone 6S camera.

### Statistical analysis

*P* values < 0.05 were considered significant for all experimental analysis. Graphpad Prism was used for statistical analysis and graph production. One-way ANOVA’s were performed on IOP measurements and retinal ganglion cell counts followed by Tukey’s post-hoc analysis to perform multiple comparison tests. Experimenters were masked to genotype during quantification of retinal ganglion cell density. Standard error of the mean was used to define error bars in all graphs. Cre negative controls from each strain did not differ from one another in IOPs or cell counts, and were thus pooled for analysis.

## Results

### Ciliary margin *Bmp4* ablation

**Fig 1:**
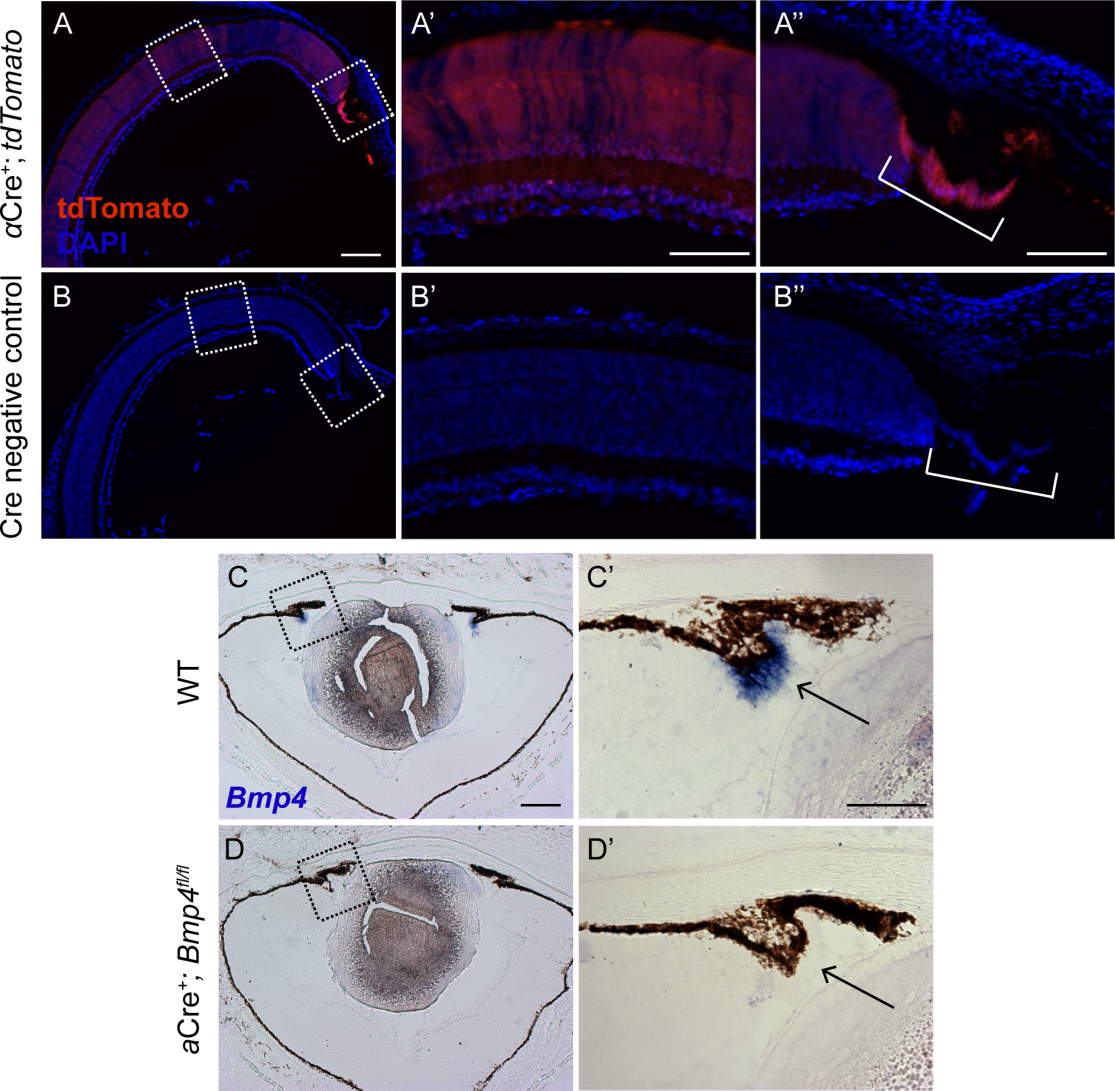
*Bmp4* is efficiently removed from the ciliary margin in conditional knockouts. Strong reporter expression is driven by *αCre* in the ciliary body of P3 mice with spotty tdTomato expression observed in the central retina **(A, A’, A”)**. Cre negative controls display no reporter expression throughout the retina **(B, B’, B”).** Sections of P1 WT and *α*Cre^+^; *Bmp4*^*fl/fl*^ mice were hybridized with a probe specific for *Bmp4*. In WT eyes, *Bmp4* mRNA (blue) is present in the developing ciliary body **(C, C’)**. No signal is detected in the ciliary body of conditional mutants **(D, D’).** *[Note*: *Bmp4 mRNA expression in the central retina is much less robust than in the ciliary body*, *and was barely detectable in these experiments*.*]* n = 3 was used per genotype per experiment. Dashed boxes in **(A)**, **(B), (C), and (D)** are enlarged in panels to the right. Consecutive sections are displayed in **C** and **C’** as well as **D** and **D’** due to processing artifacts. Scale bars represent 200μm in **(A, B, C, D)**, and 100μm in all other panels.

Chang *et al*. previously examined *Bmp4* heterozygous null mice on multiple genetic backgrounds and found that only those on a C57BL/6J background consistently presented with ASD phenotypes [12]. Thus, to ensure our mice were on the same C57BL/6J background for direct comparison to the haploinsufficient *Bmp4* mouse model, the *Bmp4* floxed allele and all Cre alleles were backcrossed between 3 and 7 generations onto the C57BL/6J genetic background prior to experimental crosses (see methods). In order to examine the role of BMP4 on anterior segment formation, without affecting lens induction [9,15,33], *Bmp4* was deleted from the ciliary margin of the optic cup using a Cre recombinase driven by the peripheral retina-specific regulatory element “α” of the murine *Pax6* gene (referred to as *α*Cre^+^; *Bmp4*^*fl/fl*^). From the onset of its expression at E10.5 through early postnatal ages, *α*Cre is expressed in the peripheral optic cup neuroepithelium [34]. To ensure this was the case in our hands, *α*Cre mice were also bred to a *Rosa26-CAGTdtomato* reporter mouse. At postnatal day (P) 3, *α*Cre-induced reporter expression was observed in the developing ciliary body (Fig 1A”) with patchy expression in the central retina (Fig 1A’) in αCre^+^; *Rosa26-CAGTdtomato*^+^; mice. No reporter expression was observed in Cre-negative littermate controls (Fig 1B–1B”).

To assess deletion efficiency in the *α*Cre^+^; *Bmp4*^*fl/fl*^ conditional mutants, *in situ* hybridization was performed using a probe specific to the floxed region of the *Bmp4* allele [31]. At P1, *Bmp4* mRNA was detected in the ciliary margin of controls (Fig 1C and 1C’), but absent in *α*Cre^+^; *Bmp4*^*fl/fl*^ conditional mutants (Fig 1D and 1D’), indicating efficient deletion by the *α*Cre allele. Additionally, genotyping analysis of tail and retinal/ciliary body DNA confirmed *Bmp4* was removed from the neuroepithelial tissue in aged conditional knockouts, but had not undergone Cre-mediated germline recombination (S1 Fig).

### Expression of BMP4 in the ciliary margin of the optic cup is not critical for anterior segment development or IOP regulation

**Fig 2:**
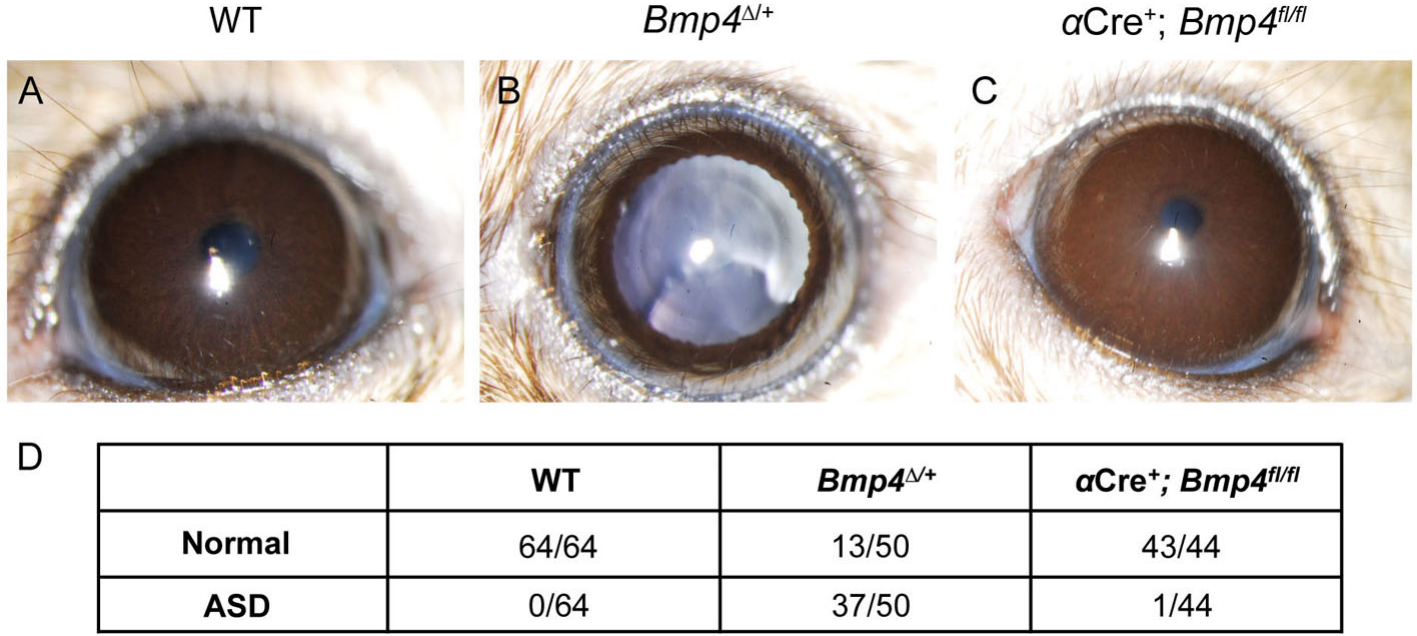
Loss of ciliary margin-derived BMP4 does not cause overt ASD. Slit lamp photographs show multiple ASD phenotypes in *Bmp4*^*Δ/+*^ mice, including iris hypoplasia shown in **(B)**, however *α*Cre^+^; *Bmp4*^*fl/fl*^ mice **(C)** are indistinguishable from controls **(A).** The total number of eyes examined for each strain is indicated in the table below **(D)**. ASD was characterized as the presence of one or more of the following: iris hypoplasia, iridocorneal adhesion, corneal opacity, corneal neovascularization, displaced pupils, cataracts, microphthalmia, and anophthalmia. One *α*Cre^+^; *Bmp4*^*fl/fl*^ mutant displayed slight pupillary displacement **(D)**, however minor ASD is also occasionally observed in wild-type animals on the C57BL/6J background.

Several groups have associated aberrant BMP4 activity with ASD phenotypes [10,12,35]. However, the spatiotemporal requirements of BMP4 for the morphogenesis of specific anterior segment structures are unknown. To compare the eyes of *α*Cre^+^; *Bmp4*^*fl/fl*^ mice to *Bmp4* heterozygous mice displaying known ASD phenotypes, a ubiquitous *CMV*Cre was crossed to the *Bmp4*^*fl/fl*^ mouse to create a germline heterozygote (referred to as *Bmp4*^*Δ/+*^). Severe ciliary body dysgenesis is commonly detected in *Bmp4* heterozygous mice [12], thus it seemed likely that BMP4 expression in the ciliary margin [14] was necessary for ciliary body development. In addition, because BMP4 is a secreted molecule, it is possible that it is required for nearby tissues such as the trabecular meshwork and cornea, which often fail to develop properly in *Bmp4*^*Δ/+*^ mice. The ASD phenotypes observed by Chang *et al*. were phenocopied in our *Bmp4*^*Δ/+*^ mice: including corneal opacity, iris hypoplasia, displaced pupils, and iridocorneal adhesions (Fig 2B and 2D). Adult *α*Cre^+^; *Bmp4*^*fl/fl*^ mice and their littermate controls (>P35) were assessed using *in vivo* slit-lamp analysis in order to detect any obvious structural or functional defects and to compare them to the eyes of similarly aged *Bmp4*^*Δ/+*^ mice. Surprisingly, while approximately 75% of *Bmp4*^*Δ/+*^ mouse eyes demonstrated ASD-associated defects, analysis revealed no overt ASD phenotypes in *α*Cre^+^; *Bmp4*^*fl/fl*^ mice as compared to controls (Fig 2A–2D).

**Fig 3:**
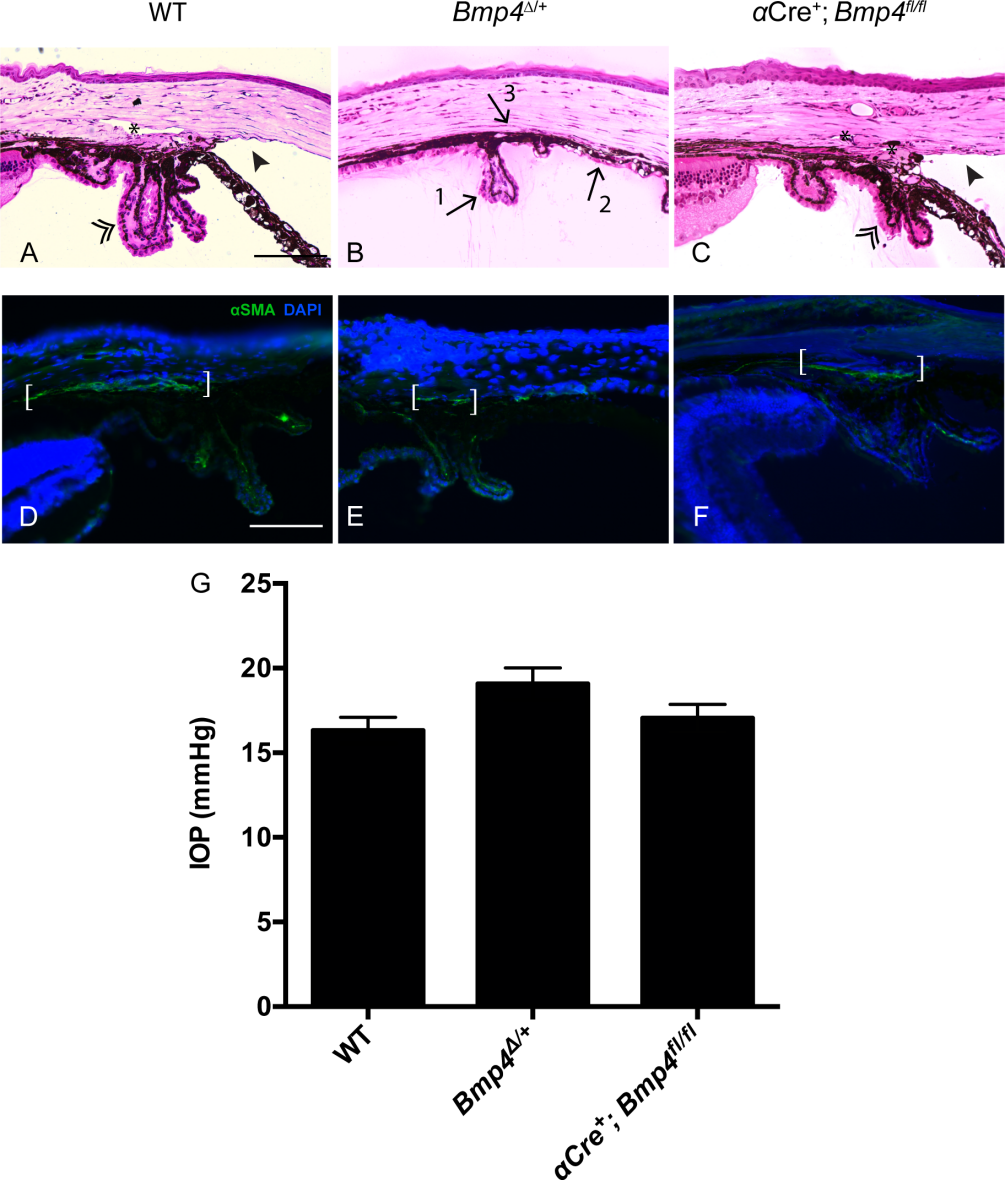
Tissues involved in IOP regulation do not require ciliary margin-derived BMP4. Semi-thin plastic sections of *Bmp4*^*Δ/+*^ eyes stained with H&E show several morphological abnormalities including a hypoplastic ciliary body (arrow #1), trabecular meshwork (arrow #2), and iris, and an absence of Schlemm’s canal (arrow #3; **B**). Note normal morphology of ciliary body (double arrowhead), iridocorneal angle (solid arrowhead), and Schlemm’s canal (asterisk) in WT as well as *α*Cre^+^; *Bmp4*^*fl/fl*^ eyes (**A, C;** n = 3 per genotype). Note there is some variability in the appearance of ciliary processes and the extent of visible Schlemm’s canal in both WT and *α*Cre^+^; *Bmp4*^*fl/fl*^ mice, however both represent normal iridocorneal angles. Extensive expression of α-SMA is observed in the trabecular meshwork (white brackets) of WT and *α*Cre^+^; *Bmp4*^*fl/fl*^ mice **(D, F)**, whereas a smaller area of immunostaining is noted in *Bmp4*^*Δ/+*^ mice (**E**; n = 3 per genotype). There was no significant difference between IOP measurements in adult mice between any of the three groups (**G**; WT n = 37, *α*Cre^+^; *Bmp4*^*fl/fl*^ n = 22m, p = 0.8237; WT n = 37, *Bmp4*^*Δ/+*^ n = 26; one-way ANOVA, p = 0.0502). Means and SEM are displayed. Scale bars in **(A-F)** represent 100μm.

To determine whether there were morphological defects that could not be detected by slit lamp examination, we prepared hematoxylin and eosin (H&E) stained semi-thin plastic sections of control, *Bmp4*^*Δ/+*^, and *α*Cre^+^; *Bmp4*^*fl/fl*^ adult mice (Fig 3A–3C). Similar to the slit lamp findings, histological examination revealed no detectable abnormalities in the *α*Cre^+^; *Bmp4*^*fl/fl*^ mutants (Fig 3C), in contrast to the *Bmp4*^*Δ/+*^ mutants, which often displayed an underdeveloped ciliary body, closed iridocorneal angle, and lack of trabecular meshwork and Schlemm’s canal (Fig 3B). To examine the trabecular meshwork more closely, frozen sections of the anterior segment were stained for α-SMA, a marker for trabecular meshwork cells [36,37]. This analysis revealed no differences in the staining between controls and *α*Cre^+^; *Bmp4*^*fl/fl*^ mutants (Fig 3D and 3F), whereas in *Bmp4*^*Δ/+*^ mutant eyes, a smaller domain of α-SMA was frequently observed (Fig 3E). The trabecular meshwork is a critical tissue for maintaining intraocular pressure (IOP) in healthy eyes. Increased IOP is often observed in children with ASD and is a leading risk factor for adult-onset open angle glaucoma [38]. Similarly, in congenital glaucoma which is frequently categorized as an ASD subtype, IOP is elevated due to a physical blockage of aqueous humor drainage [39]. Thus, in addition to histological examination of the tissues required for IOP regulation, it was also important to assess their collective functional output. IOP measurements were performed on adult *α*Cre^+^; *Bmp4*^*fl/fl*^ mice, *Bmp4*^*Δ/+*^ mice, and littermate controls from both strains (average ages +/- SEM: *α*Cre^+^; *Bmp4*^*fl/fl*^ = 3.5 +/- 0.56 mos; *Bmp4*^*Δ/+*^ mice = 3.9 +/- 0.61 mos; pooled wild-type controls = 4.1 +/- 0.48 mos). No significant differences in IOP were observed between any of the groups (Fig 3G; *α*Cre^+^; *Bmp4*^*fl/fl*^ n = 22, *Bmp4*^*Δ/+*^ n = 26, Cre^-^ controls n = 32, p>0.05). Taken together, these results indicate that deletion of *Bmp4* in the ciliary margin does not disrupt anterior segment development or function.

### Ablation of *Bmp4* from the central optic cup neuroepithelium does not disrupt posterior segment development

**Fig 4:**
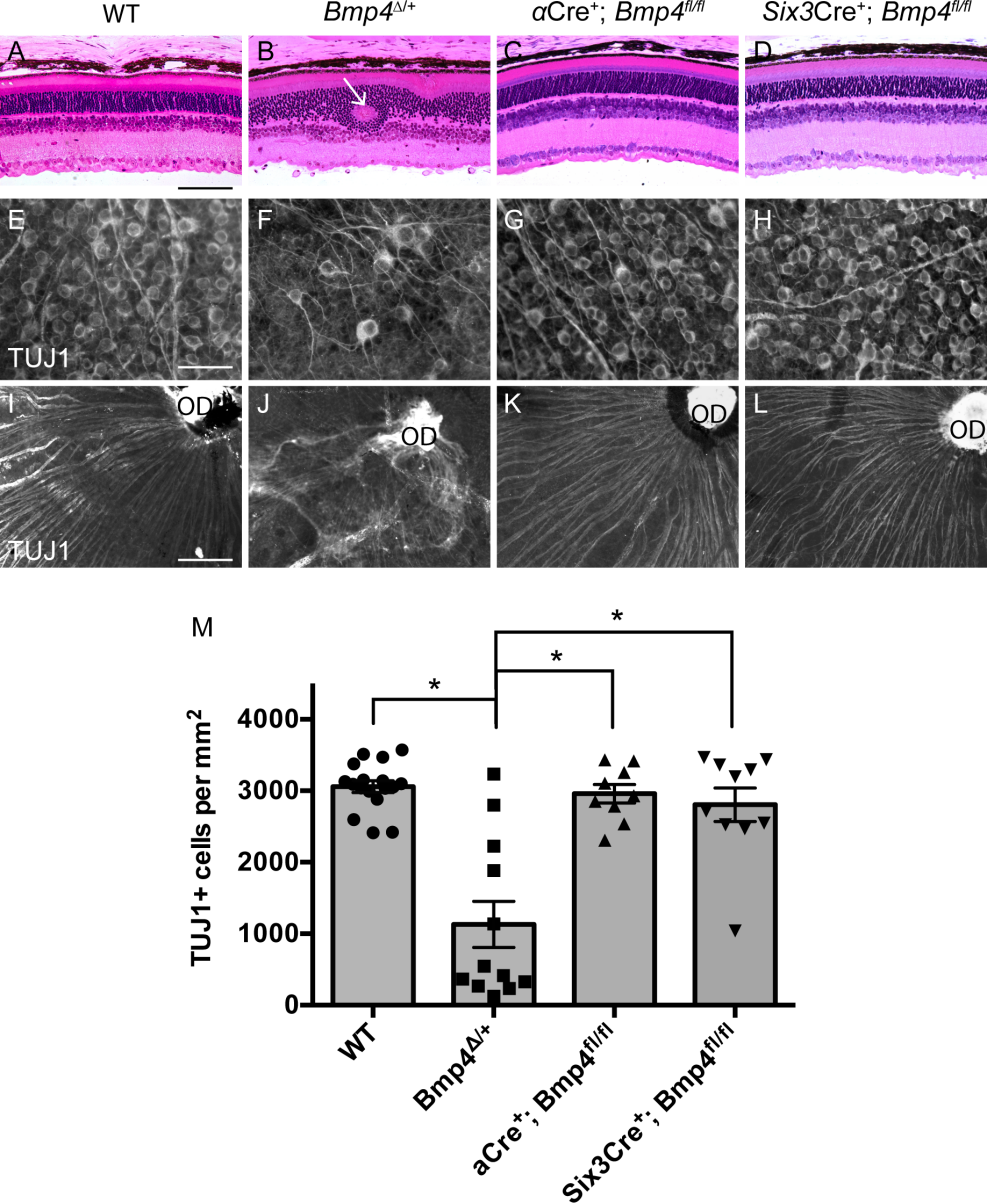
Optic cup neuroepithelium-derived BMP4 is dispensable for retinal formation. *Bmp4*^*Δ/+*^ mice display abnormalities in retinal lamination (arrow) **(B)**, retinal ganglion cell density **(F, M)**, and retinal ganglion cell axonal targeting **(J)**. Retinas of both *α*Cre^+^; *Bmp4*^*fl/fl*^ mice and *Six3*Cre^+^; *Bmp4*^*fl/fl*^ conditional mutants developed normally. Plastic sections show proper retinal lamination **(C, D).** Retinal wholemounts stained with TUJ1 reveal both normal retinal ganglion cell density **(G, H, M)** as well as proper axon targeting to the optic disc (OD) **(K, L)**. n = 4 per genotype was used for plastic sections. One-way ANOVA analysis revealed a significant difference in retinal ganglion cell counts between *Bmp4*^*Δ/+*^ mice (n = 12) and all other groups (* p<0.0001). Neither *α*Cre^+^; *Bmp4*^*fl/fl*^ (n = 9; p = 0.986) nor *Six3*Cre^+^; *Bmp4*^*fl/fl*^ (n = 10, p = 0.8026) retinal ganglion cell counts differed from WT controls (n = 17). Note: the top two data points in the *Bmp4*^*Δ/+*^ column represent retinas from eyes with normal phenotypic appearance prior to sacrifice. Means and SEM are displayed. Scale bars in **(A-L)** represent 100μm.

**Fig 5:**
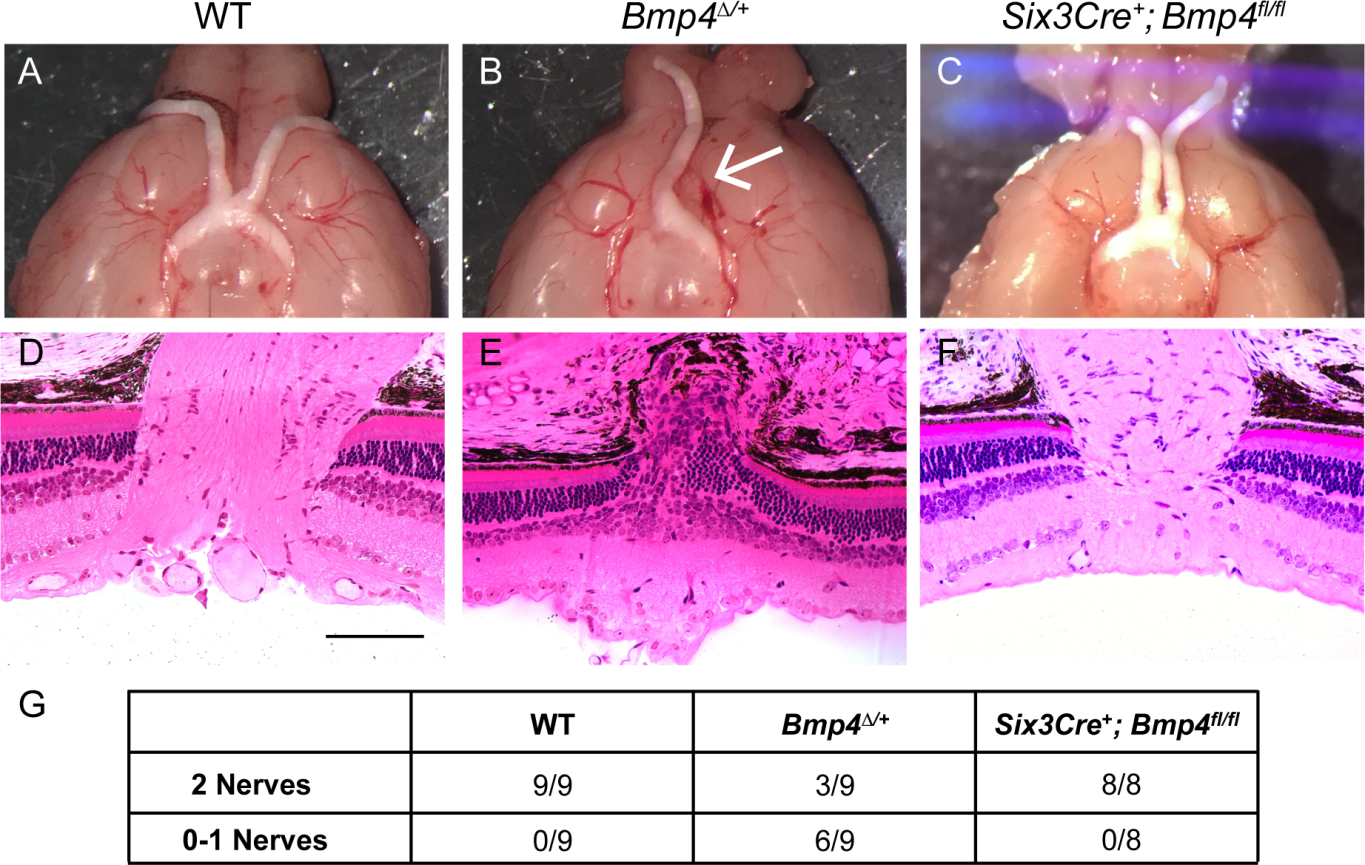
Central optic cup neuroepithelium-derived BMP4 is not required for optic nerve formation. Control animals display normal gross optic nerve and optic chiasm morphology **(A)**, as well proper axonal exit at the optic nerve head shown in plastic sections **(D)**. *Bmp4*^*Δ/+*^ mice are often missing one (arrow) or both nerves **(B)**, and retinal ganglion cell axons do not properly exit the eye **(E)**. Optic nerves of *Six3*Cre^+^; *Bmp4*^*fl/fl*^ mice appear indistinguishable from controls **(C, F)**. The total number of brains examined for each strain is shown in the table below **(G)**. At least 4 eyes per genotype were sectioned for histology. Scale bars in **(D-F)** represent 100μ.

Although BMP4 did not appear to be involved in formation of the anterior segment, it remained possible that because there have been reports of BMP4 expression within the retina at multiple time points, and because *Bmp4*^*Δ/+*^ mutants also display posterior defects, BMP4 derived from the central optic cup neuroepithelium could be necessary for retinal differentiation and optic nerve development [13,40]. Since *α*Cre^+^ has limited recombination efficiency in the central retina (Fig 1A’ and [33,41]), we additionally employed *Six3*Cre (regularly used by our group [29,32,42] and many others for retinal-specific conditional deletion) to remove *Bmp4* in the central optic cup neuroepithelium (S2 Fig). This conditional knockout (referred to as *Six3*Cre^+^; *Bmp4*^*fl/fl*^) also provided further confirmation of the aforementioned anterior segment findings, as it yields modest deletion of *Bmp4* mRNA in the ciliary margin (S2 Fig). Similar to our anterior segment methodology, adult *α*Cre^+^; *Bmp4*^*fl/fl*^ and *Six3*Cre^+^; *Bmp4*^*fl/fl*^ mice were examined to detect any retinal defects in the mature posterior segment. Adult mice heterozygous for the *Bmp4* null mutation display abnormalities in retinal lamination, optic nerve formation, and retinal vasculature [12]. We found similar abnormalities within our *Bmp4*^*Δ/+*^ mice, which often exhibited disruptions in all retinal layers (Fig 4B) and optic nerve malformation (Fig 5B and 5E). We also found a significant decrease in retinal ganglion cell density (Fig 4F and 4M; *Bmp4*^*Δ/+*^ n = 12, Cre^-^ controls n = 17, p<0.0001) and misdirected axon targeting (Fig 4J) in the majority of heterozygotes, not previously reported.

Unlike the *Bmp4*^*Δ/+*^ mutants, however, the posterior segments of both *α*Cre^+^; *Bmp4*^*fl/fl*^ mice and *Six3*Cre^+^; *Bmp4*^*fl/fl*^ mice displayed normal morphology. In both conditional mutants, H&E stained plastic sections showed proper retinal lamination (Fig 4C and 4D), retinal wholemounts stained with TUJ1 revealed normal retinal ganglion cell density (Fig 4G, 4H and 4M; *α*Cre^+^; *Bmp4*^*fl/fl*^ n = 9, *Six3*Cre^+^; *Bmp4*^*fl/fl*^ n = 10, Cre^-^ controls n = 17, p>0.05), and retinal ganglion cell axons were appropriately targeted to the optic disc (Fig 4K and 4L).

To examine the optic nerve morphology after exiting the eye, brains from each group were removed to examine the optic nerves in wholemount (n = 8 per genotype). *Bmp4*^*Δ/+*^ mice often show an absence of one or both optic nerves (Fig 5B and 5G), whereas in contrast, *Six3*Cre^+^; *Bmp4*^*fl/fl*^ mice displayed normal optic nerve formation (Fig 5C and 5G). The eyes corresponding to each nerve were analyzed by plastic histology to more closely assess the configuration of the optic nerve head. The axons of retinal ganglion cells appropriately coalesced to form the optic nerve and exit the eye in controls as well as in *Six3*Cre^+^; *Bmp4*^*fl/fl*^ mutants (Fig 5D and 5F). However, the retinal ganglion cell axons of some *Bmp4*^*Δ/+*^ mice failed to establish a normal optic nerve and thus did not exit the eye (Fig 5E). Three of the nine brains examined, however, displayed two normal optic nerves (Fig 5G), as seen in both wholemount and section (images not shown).

**Fig 6:**
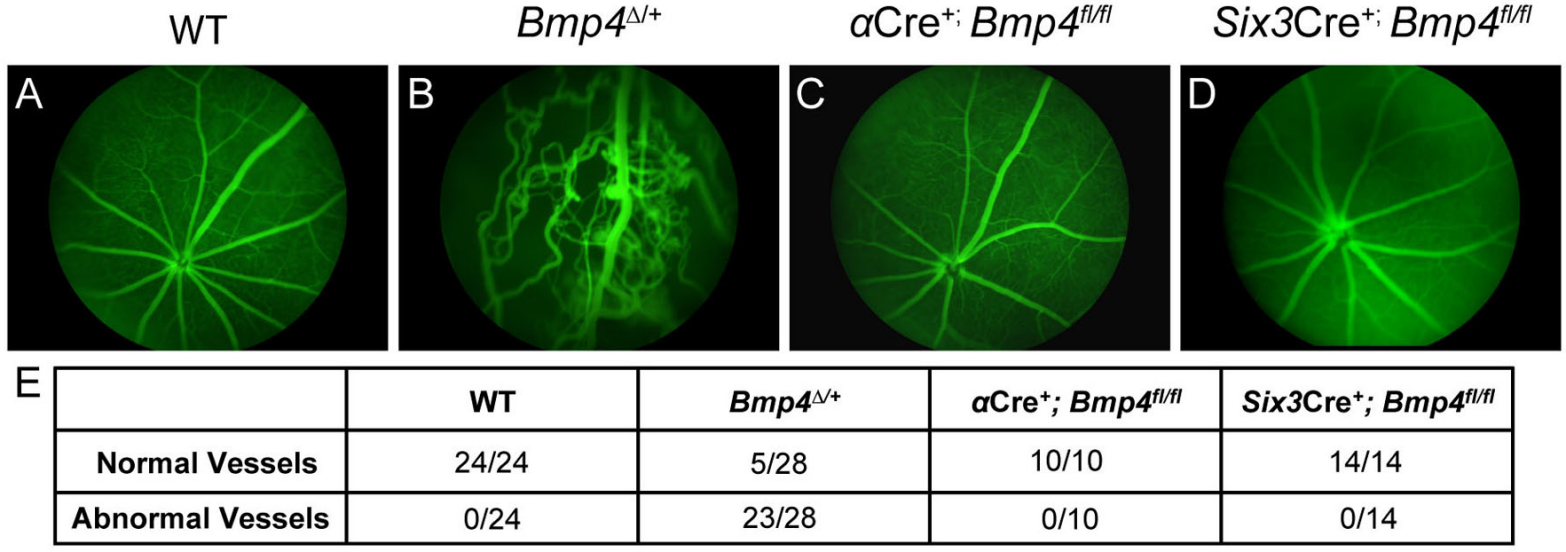
The retinal vasculature is properly organized following loss of optic cup neuroepithelium-derived BMP4. Fundus exams using fluorescein angiography reveal highly abnormal vasculature patterning in *Bmp4*^*Δ/+*^ mice **(B)**, however all *α*Cre^+^; *Bmp4*^*fl/fl*^ and *Six3*Cre^+^; *Bmp4*^*fl/fl*^ mutants examined display normal vasculature formation **(C, D)**. The total number of eyes examined for each strain is shown in the table below **(E)**. Abnormal vessels were defined as blood leakage, irregularly arranged retinal capillaries, and vessel protrusion into the vitreous body.

An additional cohort of mice underwent fluorescein angiography to visualize retinal vasculature patterning. Fundus images revealed several abnormalities in *Bmp4*^*Δ/+*^ mice, including vessel leakage, vessel protrusion into the vitreous, and overlapping vessels (Fig 6B), previously reported by Chang *et al* [12]. The vasculature of *α*Cre^+^; *Bmp4*^*fl/fl*^ and *Six3*Cre^+^; *Bmp4*^*fl/fl*^ mutants was indistinguishable from controls (Fig 6C–6E).

### Anterior and posterior dysgenesis are linked in *Bmp4* heterozygous mice

**Fig 7:**
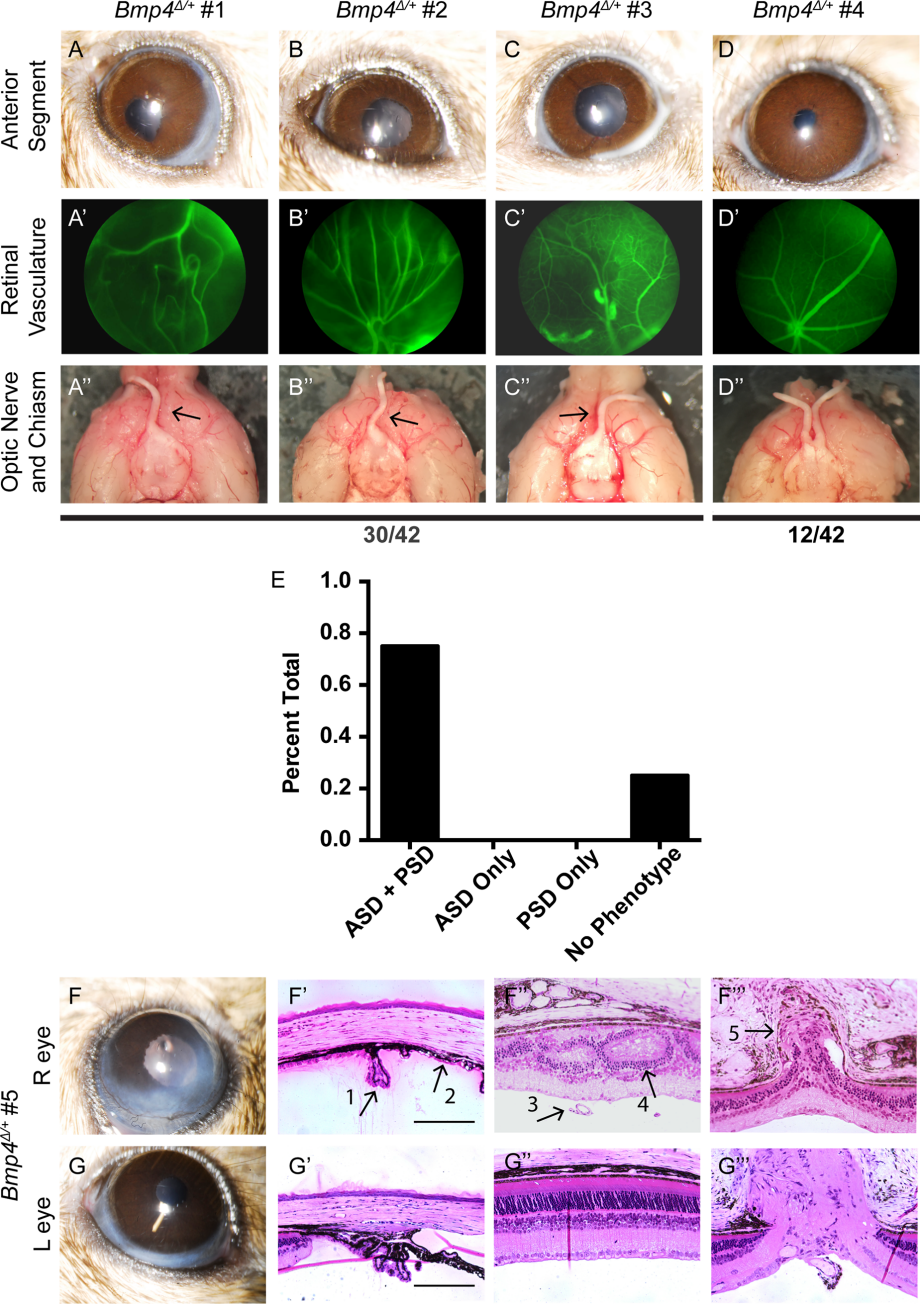
Anterior and posterior abnormalities occur concurrently or not at all. Slit lamp images, fundus exams, and gross optic nerve morphology was assessed sequentially in 42 eyes of *Bmp4*^*Δ/+*^ mice. 30/42 eyes displayed each of the following: ASD, abnormal vasculature, and absence of an optic nerve **(A-C, A’-C’, A”-C”)**. The remaining 12 eyes appeared normal in each category **(D-D”)**. None of the eyes examined presented with only anterior segment dysgenesis or only posterior segment dysgenesis **(E)**. An additional cohort (n = 6 eyes; 3 phenotypically abnormal and 3 with phenotypically normal based upon *in vivo* imaging) was assessed with plastic histology following slit lamp and fundus examination. Representative images from the same animal illustrate that the phenotypic presentation of the anterior segment **(F, G)** always predicts the presence or absence of morphological disruption in the posterior segment **(F”, F”‘, G”, G”‘)**. Arrows represent: hypoplastic ciliary body (#1, **F’)**, iridocorneal adhesion (#2, **F’**), persistent hyaloid vasculature (#3, **F”**), disrupted retinal lamination (#4, **F”**), and obstructed optic nerve exit (#5, **F”‘**). Scale bars represent 100μ.

Several *Bmp4*^*Δ/+*^ mice in both the anterior segment and posterior segment analyses appeared to be anatomically and quantitatively normal. This raised the interesting question of whether the anterior and posterior phenotypes in the *Bmp4*^*Δ/+*^ mice correlate with each other, or alternatively occur independently. Thus, we assessed an additional cohort of *Bmp4*^*Δ/+*^ eyes for anterior and posterior phenotypes via slit lamp, fluorescein angiography, and gross histology on a per eye basis. Intriguingly, we found a 100% correlation between anterior and posterior phenotypes in *Bmp4* heterozygous mice. Each eye examined either had both anterior and posterior segment abnormalities, or had no defects in either segment (Fig 7). Slit lamp images displaying ASD phenotypes such as iris hypoplasia, pupil displacement, and corneal opacity (Fig 7A–7C) were always predictive of abnormal retinal vasculature observed in fundus examination (Fig 7A’–7C’), as well as the absence of that optic nerve (Fig 7A”–7C”). Conversely, animals with normal anterior segments *in vivo* (Fig 7D) displayed proper retinal vasculature patterning (Fig 7D’) and the presence of that optic nerve (Fig 7D”). An additional cohort of *Bmp4*^*Δ/+*^ animals was assessed with both *in vivo* imaging and plastic histology, further confirming that anterior and posterior phenotypes are correlated, with dysgenesis often occurring in only one eye of a mutant (Fig 7F–7G”‘). These results suggest a linkage between anterior and posterior defects, and may indicate a common origin of these abnormalities.

## Discussion

The present study has demonstrated that from the optic cup stage on, retinal neuroepithelium-derived BMP4 does not play a critical role in the development of the mouse eye. We analyzed both anterior and posterior segment tissues in the eyes of *α*Cre^+^; *Bmp4*^*fl/fl*^ and *Six3*Cre^+^; *Bmp4*^*fl/fl*^ mutant mice. Despite reported embryonic BMP4 expression in the ciliary margin and retinal neuroepithelium throughout development, as well as severe dysgenesis in *Bmp4* haploinsufficient mice, targeted *Bmp4* ablation from the peripheral and central optic cup neuroepithelium had no major deleterious effects on the formation of the anterior segment, retina, optic nerve, or retinal vasculature.

Previously, we found evidence suggesting BMP signaling was involved in ciliary body formation. In outer ciliary epithelium-specific *Notch2* knockout mice, the ciliary body failed to develop and increased levels of BMP inhibitor mRNA were noted [43]. Furthermore, the addition of a *Chrdl1*, a pan BMP inhibitor, resulted in misfolding of the ciliary epithelium [43]. The most likely ligand to mediate this signaling is BMP4, given its expression in the embryonic ciliary margin and developing ciliary body [7,8,14,20,22]. In addition, loss of one copy of *Bmp4* was reported to cause defects in the ciliary body [12]. To our surprise however, deletion of both copies of *Bmp4* specifically within the ciliary margin of the optic cup did not affect ciliary body formation. This finding does not necessarily contradict previous evidence suggesting BMP signaling is required for proper ciliary body development [8,14,43]—only that optic cup-derived BMP4 is not the required molecular mediator.

In order to assess whether other anterior segment structures might be affected by ciliary margin-specific loss of BMP4, we examined the gross morphology of the cornea, trabecular meshwork, and Schlemm’s canal. Though not specifically expressed in these tissues, the secreted nature of BMP4 could elicit critical downstream effects on proximate tissues. However, each of these structures was histologically normal in *α*Cre^+^; *Bmp4*^*fl/fl*^ mutants. No histological defects were observed in the cornea, lens, or iris, consistent with the slit lamp analysis *in vivo*. Additionally, functional readout of the structures required for IOP regulation (ciliary body, trabecular meshwork, and Schlemm’s canal) showed no statistical difference between mutants and controls, or among mutants over time. This too correlated with histological findings—evidenced by normal morphology and a standard expression pattern of the mature trabecular meshwork marker, α-SMA, in *α*Cre^+^; *Bmp4*^*fl/fl*^ mice. However, as histological analysis was not performed at the EM level, it is possible that subtle defects in the iridocorneal angle tissues were present but not detected. It is also worth considering that the anterior segment structures in *α*Cre^+^; *Bmp4*^*fl/fl*^ and *Six3*Cre^+^; *Bmp4*^*fl/fl*^ mice may be more susceptible to cellular stress or aging due to subtle developmental defects and/or a requirement of ciliary body-derived BMP4 to maintain proper function. Thus, aged mice might have a greater propensity for impaired aqueous outflow and ocular hypertension. Since all mice examined were young (3–4 months old) we cannot rule out this possibility. In the future, it will be important to age these mice to fully assess the role of ciliary body-derived BMP4 in anterior segment physiology.

It was also surprising to find a complete lack of retinal dysgenesis in both *α*Cre^+^; *Bmp4*^*fl/fl*^ and *Six3*Cre^+^; *Bmp4*^*fl/fl*^ adult mice, given the extensive defects seen in *Bmp4* haploinsufficient mutants. BMP4 is expressed in retinal neuroblasts, and levels of retinal *Bmp4* mRNA are drastically reduced in a mouse model of photoreceptor degeneration [13,40]. We showed—consistent with Chang *et al* [12]—that somatic *Bmp4* haploinsufficiency resulted in several retinal defects including aberrant retinal lamination, optic nerve abnormalities, and improper retinal vasculature arrangement. Unreported by Chang *et al* [12] however, was our discovery of a quantified reduction in retinal ganglion cell density and impaired axon targeting in the majority of *Bmp4* heterozygotes. However, upon analysis of both conditional knockouts, no retinal irregularities were detected. As with the anterior segment analysis, aged and/or stressed animals were not examined in this study. Therefore, it remains an open question whether retinal phenotypes could emerge following stressors such as aging, neuronal injury, or the presence of disease that affects RGCs (e.g. diabetic retinopathy or glaucoma).

It is possible that optic cup ablation of *Bmp4* can be compensated for by BMP2 or BMP7, as these ligands have similar expression patterns in the optic cup and ciliary margin respectively [13,22,44,45]. BMP7 supplementation can rescue ciliary body defects elicited by addition of the BMP inhibitor, Noggin, and thus may play a distinct or cooperative role in ciliary body morphogenesis [44]. Additionally, compound heterozygous mice for *Bmp4* and *Bmp2* present with microphthalmia and retinal degeneration [46]. This possibility seems unlikely, however, considering genetic compensation by other BMP ligands does not prevent the dysgenesis observed in optic vesicle-depleted *Bmp4* conditional mutants or in *Bmp4*^*Δ/+*^ mice [9,12].

Genetic background can also play an important role in phenotypic penetrance [47–49]. Because Chang *et al* determined that ocular abnormalities in *Bmp4*^*Δ/+*^ mice were most consistently present on the C57BL/6J background, we sought to ensure the background strain was consistent when comparing our conditional and haploinsufficient mutants. All *α*Cre^+^; *Bmp4*^*fl/fl*^, *Six3*Cre^+^; *Bmp4*^*fl/fl*^, and *Bmp4*^*Δ/+*^ mice examined in the present study were backcrossed for at least three generations onto C57BL/6J. Since no anterior or posterior segment abnormalities were observed in the conditional mutants, we are confident that the developmental ASD and retinal defects detected in *Bmp4* haploinsufficient mice on the C57BL/6J background are not due to the loss of optic cup neuroepithelium-derived BMP4. Interestingly, Van der Merwe & Kidson showed that nearly half of the *Bmp4* heterozygous null mice they examined on an ICR background (a strain absent from Chang and colleagues’ study) displayed missing portions of Schlemm’s canal [35], suggesting that ASD phenotypes caused by *Bmp4* mutations are not exclusive to C57BL/6J mice. The identification of strain-dependent phenotypic penetrance, as well as possible genetic modifiers of BMP4, requires additional study.

Our data rule out an essential cell-autonomous role of BMP4 in the optic cup, despite well-documented expression in the ciliary margin and retinal neuroepithelium [7,13,20]. The lack of an ocular phenotype in our conditional mutants is surprising given that loss of only one copy of *Bmp4* causes widespread ocular defects in the heterozygous mice. Previous studies have demonstrated that BMP4 is required at the optic vesicle stage for retinal specification and/or lens-induction [9]. Thus, the ocular phenotypes arising in the *Bmp4* heterozygous mutants could result from a reduction of BMP4 at the optic vesicle stage, or from a diminished source of BMP4 outside the optic cup. It is currently unclear which tissues outside the optic cup could be providing a critical source of BMP4. It is possible that subtle defects in lens induction arising from insufficient expression of BMP4 intrinsically in the lens or extrinsically in the optic vesicle might contribute to the later anterior and posterior segment abnormalities in the affected *Bmp4*^*Δ/+*^ eyes; however, we did not observe any overt lens malformation in our analysis of these mice. BMP4 is also expressed in the ocular mesenchyme [7,22,50], which is well established in contributing to ASD phenotypes (reviewed in [2]), although how mesenchymal BMP4 could affect the posterior segment is less clear. Additional work should investigate the role of retinal vascular endothelial cells as a critical source of BMP4 as well [51]. Overall our studies have established that BMP4 is not required cell autonomously in the optic cup for subsequent physiological eye development. Further studies should reveal whether there is a yet unidentified source of BMP4 that is critical for ocular morphogenesis.

## Supporting information

S1 Fig PCR validation. Germline deletion does not occur in conditional knockouts. Genotyping results show presence or absence of *Cre*, *Bmp4flox*, *WT*, and *Bmp4* recombined alleles in DNA extracted from the tail and the retina of adult WT mice, *α*Cre^+^; *Bmp4*^*fl/fl*^ mice, *Six3*Cre^+^; *Bmp4*^*fl/fl*^ mice and mice heterozygous for a null allele of *Bmp4*. Note the absence of the deleted band in tail DNA from *α*Cre^+^; *Bmp4*^*fl/fl*^ mice and *Six3*Cre^+^; *Bmp4*^*fl/fl*^ mice, indicating germline recombination had not occurred.
(TIF)

S2 Fig *Six3*Cre recombination efficiency. *Bmp4* is efficiently removed from the central retina in *Six3*Cre-mediated conditional knockouts. TdTomato reporter expression driven by *Six3*Cre is detected throughout the central retina **(A, A’)** and sporadically in the peripheral retina and ciliary body **(A, A”)** in P3 mice. No reporter expression is seen in Cre negative controls **(B-B”)**. Brackets denote ciliary body. (**C-D”)** Sections of P1 WT and *Six3*Cre^+^; *Bmp4*^*fl/fl*^ mice were hybridized with a probe specific for *Bmp4*. In WT eyes, *Bmp4* mRNA is present at low levels throughout the central retina **(C, C’)** and robustly in ciliary body **(C”)**. *Bmp4* mRNA is absent in the retina of *Six3*Cre^+^; *Bmp4*^*fl/fl*^ mice **(D, D’)** and reduced in the ciliary body **(D”)**. Arrows point to ciliary body. n = 3 per genotype per experiment. Dashed boxes in **(A)**, **(B), (C), and (D)** are enlarged in panels to the right. Different sections from the same eye are shown in **C-C”** as well as **D**-**D”** due to sectioning/processing artifacts. Scale bars represent 100μm in all panels.
(TIF)

## References

[1] Chow RL, Lang RA. Early eye development in vertebrates. Annu Rev Cell Dev Biol. 2001;17: 255–296. doi: 10.1146/annurev.cellbio.17.1.255 11687490 10.1146/annurev.cellbio.17.1.255

[2] Cvekl A, Tamm ER. Anterior eye development and ocular mesenchyme: new insights from mouse models and human diseases. Bioessays. 2004;26: 374–386. doi: 10.1002/bies.20009 15057935 10.1002/bies.20009 PMC2094210

[3] Graw J. The genetic and molecular basis of congenital eye defects. Nat Rev Genet. 2003;4: 876–888. doi: 10.1038/nrg1202 14634635 10.1038/nrg1202

[4] Ito YA, Walter MA. Genomics and anterior segment dysgenesis: a review. Clin Exp Ophthalmol. 2013;42: 13–24. doi: 10.1111/ceo.12152 24433355 10.1111/ceo.12152

[5] Reis LM, Semina EV. Genetics of anterior segment dysgenesis disorders. Curr Opin Ophthalmol. 2011;22: 314–324. doi: 10.1097/ICU.0b013e328349412b 21730847 10.1097/ICU.0b013e328349412b PMC3558283

[6] Wang RN, Green J, Wang Z, Deng Y, Qiao M, Peabody M, et al Bone Morphogenetic Protein (BMP) signaling in development and human diseases. Genes Dis. 2014;1: 87–105. doi: 10.1016/j.gendis.2014.07.005 25401122 10.1016/j.gendis.2014.07.005 PMC4232216

[7] Furuta Y, Hogan BL. BMP4 is essential for lens induction in the mouse embryo. Genes Dev. 1998;12: 3764–3775. 9851982 10.1101/gad.12.23.3764 PMC317259

[8] Zhao S, Chen Q, Hung F-C, Overbeek PA. BMP signaling is required for development of the ciliary body. Development. 2002;129: 4435–4442. 12223402 10.1242/dev.129.19.4435

[9] Huang J, Liu Y, Oltean A, Beebe DC. Bmp4 from the optic vesicle specifies murine retina formation. Dev Biol. 2015; 402: 119–126. doi: 10.1016/j.ydbio.2015.03.006 25792196 10.1016/j.ydbio.2015.03.006 PMC4523094

[10] Bakrania P, Efthymiou M, Klein JC, Salt A, Bunyan DJ, Wyatt A, et al Mutations in BMP4 cause eye, brain, and digit developmental anomalies: overlap between the BMP4 and hedgehog signaling pathways. Am J Hum Genet. 2008;82: 304–319. doi: 10.1016/j.ajhg.2007.09.023 18252212 10.1016/j.ajhg.2007.09.023 PMC2427285

[11] Hayashi S, Okamoto N, Makita Y, Hata A, Imoto I, Inazawa J. Heterozygous deletion at 14q22.1-q22.3 including the BMP4 gene in a patient with psychomotor retardation, congenital corneal opacity and feet polysyndactyly. Am J Med Genet. 2008;146A: 2905–2910. doi: 10.1002/ajmg.a.32519 18925664 10.1002/ajmg.a.32519

[12] Chang B, Smith RS, Peters M, Savinova OV, Hawes NL, Zabaleta A, et al Haploinsufficient Bmp4 ocular phenotypes include anterior segment dysgenesis with elevated intraocular pressure. BMC Genet. 2001;2: 18 doi: 10.1186/1471-2156-2-18 11722794 10.1186/1471-2156-2-18 PMC59999

[13] Du Y, Xiao Q, Yip HK. Regulation of retinal progenitor cell differentiation by Bone Morphogenetic Protein 4 is mediated by the Smad/Id cascade. Invest Ophthalmol Vis Sci. 2010;51: 3764–3773. doi: 10.1167/iovs.09-4906 20130285 10.1167/iovs.09-4906

[14] Napier HRL, Kidson SH. Molecular events in early development of the ciliary body: a question of folding. Exp Eye Res. 2007;84: 615–625. doi: 10.1016/j.exer.2006.07.012 16959249 10.1016/j.exer.2006.07.012

[15] Cvekl A, Ashery-Padan R. The cellular and molecular mechanisms of vertebrate lens development. Development. 2014;141: 4432–4447. doi: 10.1242/dev.107953 25406393 10.1242/dev.107953 PMC4302924

[16] Adler R, Canto-Soler MV. Molecular mechanisms of optic vesicle development: complexities, ambiguities and controversies. Dev Biol. 2007;305: 1–13. doi: 10.1016/j.ydbio.2007.01.045 17335797 10.1016/j.ydbio.2007.01.045 PMC1927083

[17] Klimova L, Lachova J, Machon O, Sedlacek R, Kozmik Z. Generation of mRx-Cre transgenic mouse line for efficient conditional gene deletion in early retinal progenitors. PLoS ONE. 2013;8(5): e63029 doi: 10.1371/journal.pone.0063029 23667567 10.1371/journal.pone.0063029 PMC3646923

[18] Swindell EC, Bailey TJ, Loosli F, Liu C, Amaya-Manzanares F, Mahon KA, et al Rx-Cre, a tool for inactivation of gene expression in the developing retina. genesis. 2006;44: 361–363. doi: 10.1002/dvg.20225 16850473 10.1002/dvg.20225

[19] Ashery-Padan R, Marquardt T, Zhou X, Gruss P. Pax6 activity in the lens primordium is required for lens formation and for correct placement of a single retina in the eye. Genes Dev. 2000;14: 2701–2711. 11069887 10.1101/gad.184000 PMC317031

[20] Huang J, Liu Y, Filas B, Gunhaga L, Beebe DC. Negative and positive auto-regulation of BMP expression in early eye development. Dev Biol. 2015;407: 256–264. doi: 10.1016/j.ydbio.2015.09.009 26407529 10.1016/j.ydbio.2015.09.009 PMC4663133

[21] Fuhrmann S. Eye morphogenesis and patterning of the optic vesicle. Curr Top Dev Biol. 2010;93: 61–84. doi: 10.1016/B978-0-12-385044-7.00003-5 20959163 10.1016/B978-0-12-385044-7.00003-5 PMC2958684

[22] Dudley AT, Robertson EJ. Overlapping expression domains of bone morphogenetic protein family members potentially account for limited tissue defects in BMP7 deficient embryos. Dev Dyn. 1997;208: 349–362. doi: 10.1002/(SICI)1097-0177(199703)208:3<349::AID-AJA6>3.0.CO;2-I 9056639 10.1002/(SICI)1097-0177(199703)208:3<349::AID-AJA6>3.0.CO;2-I

[23] Matsushima D, Heavner W, Pevny LH. Combinatorial regulation of optic cup progenitor cell fate by SOX2 and PAX6. Development. 2011;138: 443–454. doi: 10.1242/dev.055178 21205789 10.1242/dev.055178 PMC3014633

[24] Marquardt T, Gruss P. Generating neuronal diversity in the retina: one for nearly all. Trends Neurosci. 2002;25: 32–38. 11801336 10.1016/s0166-2236(00)02028-2

[25] Liu W, Selever J, Wang D, Lu M-F, Moses KA, Schwartz RJ, et al Bmp4 signaling is required for outflow-tract septation and branchial-arch artery remodeling. Proc Natl Acad Sci USA. 2004;101: 4489–4494. doi: 10.1073/pnas.0308466101 15070745 10.1073/pnas.0308466101 PMC384774

[26] Furuta Y, Lagutin O, Hogan BL, Oliver GC. Retina- and ventral forebrain-specific Cre recombinase activity in transgenic mice. genesis. 2000;26: 130–132. 10686607

[27] Schwenk F, Baron U, Rajewsky K. A cre-transgenic mouse strain for the ubiquitous deletion of loxP-flanked gene segments including deletion in germ cells. Nucleic Acids Res. 1995;23: 5080–5081. 8559668 10.1093/nar/23.24.5080 PMC307516

[28] Madisen L, Zwingman TA, Sunkin SM, Oh SW, Zariwala HA, Gu H, et al A robust and high-throughput Cre reporting and characterization system for the whole mouse brain. Nat Neurosci. 2009;13: 133–140. doi: 10.1038/nn.2467 20023653 10.1038/nn.2467 PMC2840225

[29] Fernandes KA, Harder JM, John SW, Shrager P, Libby RT. DLK-dependent signaling is important for somal but not axonal degeneration of retinal ganglion cells following axonal injury. Neurobiol Dis. 2014;69: 108–116. doi: 10.1016/j.nbd.2014.05.015 24878510 10.1016/j.nbd.2014.05.015 PMC4099422

[30] Bulchand S, Subramanian L, Tole S. Dynamic spatiotemporal expression of LIM genes and cofactors in the embryonic and postnatal cerebral cortex. Dev Dyn. 2003;226: 460–469. doi: 10.1002/dvdy.10235 12619132 10.1002/dvdy.10235

[31] Jia S, Zhou J, Gao Y, Baek J-A, Martin JF, Lan Y, et al Roles of Bmp4 during tooth morphogenesis and sequential tooth formation. Development. 2013;140: 423–432. doi: 10.1242/dev.081927 23250216 10.1242/dev.081927 PMC3597212

[32] Harder JM, Fernandes KA, Libby RT. The Bcl-2 family member BIM has multiple glaucoma-relevant functions in DBA/2J mice. Sci Rep. 2012;2: 530 doi: 10.1038/srep00530 22833783 10.1038/srep00530 PMC3404412

[33] C Cai Z, Feng G-S, Zhang X. Temporal requirement of the protein tyrosine phosphatase Shp2 in establishing the neuronal fate in early retinal development. J Neurosci. 2010;30: 4110–4119. doi: 10.1523/JNEUROSCI.4364-09.2010 20237281 10.1523/JNEUROSCI.4364-09.2010 PMC2845916

[34] Marquardt T, Ashery-Padan R, Andrejewski N, Scardigli R, Guillemot F, Gruss P. Pax6 is required for the multipotent state of retinal progenitor cells. Cell. 2001;105: 43–55. 11301001 10.1016/s0092-8674(01)00295-1

[35] van der Merwe EL, Kidson SH. Wholemount imaging reveals abnormalities of the aqueous outflow pathway and corneal vascularity in Foxc1 and Bmp4 heterozygous mice. Exp Eye Res. 2016;146: 293–303. doi: 10.1016/j.exer.2016.04.003 27068508 10.1016/j.exer.2016.04.003

[36] de Kater AW, Shahsafaei A, Epstein DL. Localization of smooth muscle and nonmuscle actin isoforms in the human aqueous outflow pathway. Inves Ophthalmol Vis Sci. 1992;33: 424–429. 1740375

[37] MinHee K Ko J CHT. Contractile markers distinguish structures of the mouse aqueous drainage tract. Mol Vis. 2013;19: 2561–2570. 24357924 PMC3867161

[38] Keller KE, Acott TS. The juxtacanalicular region of ocular trabecular meshwork: a tissue with a unique extracellular matrix and specialized function. J Ocul Biol. 2013;1: 3 24364042 PMC3867143

[39] Ko F, Papadopoulos M, Khaw PT. Primary congenital glaucoma. Prog Brain Res. 2015;221: 177–189. doi: 10.1016/bs.pbr.2015.06.005 26518078 10.1016/bs.pbr.2015.06.005

[40] Mathura JR, Jafari N, Chang JT, Hackett SF, Wahlin KJ, Della NG, et al Bone morphogenetic proteins-2 and -4: negative growth regulators in adult retinal pigmented epithelium. Invest Ophthalmol Vis Sci. 2000;41: 592–600. 10670493

[41] Gueta K, David A, Cohen T, Menuchin-Lasowski Y, Nobel H, Narkis G, et al The stage-dependent roles of Ldb1 and functional redundancy with Ldb2 in mammalian retinogenesis. Development. 2016;143: 4182–4192. doi: 10.1242/dev.129734 27697904 10.1242/dev.129734 PMC5117211

[42] Fernandes KA, Harder JM, Kim J, Libby RT. JUN regulates early transcriptional responses to axonal injury in retinal ganglion cells. Exp Eye Res. 2014;112: 106–117. doi: 10.1016/j.exer.2013.04.021 23648575 10.1016/j.exer.2013.04.021 PMC3700614

[43] Zhou Y, Tanzie C, Yan Z, Chen S, Duncan M, Gaudenz K, et al Notch2 regulates BMP signaling and epithelial morphogenesis in the ciliary body of the mouse eye. Proc Natl Acad Sci U.S.A. 2013;110: 8966–8971. doi: 10.1073/pnas.1218145110 23676271 10.1073/pnas.1218145110 PMC3670365

[44] Hung F-C, Zhao S, Chen Q, Overbeek PA. Retinal ablation and altered lens differentiation induced by ocular overexpression of BMP7. Vision Res. 2002;42: 427–438. 11853758 10.1016/s0042-6989(01)00242-5

[45] Hussein KA, Choksi K, Akeel S, Ahmad S, Megyerdi S, El-Sherbiny M, et al Bone morphogenetic protein 2: a potential new player in the pathogenesis of diabetic retinopathy. Exp Eye Res. 2014;125: 79–88. doi: 10.1016/j.exer.2014.05.012 24910902 10.1016/j.exer.2014.05.012 PMC4122600

[46] Uchimura T, Komatsu Y, Tanaka M, McCann KL, Mishina Y. Bmp2 and Bmp4 genetically interact to support multiple aspects of mouse development including functional heart development. genesis. 2009;47: 374–384. doi: 10.1002/dvg.20511 19391114 10.1002/dvg.20511 PMC2847484

[47] Kiernan AE, Li R, Hawes NL, Churchill GA, Gridley T. Genetic background modifies inner ear and eye phenotypes of Jag1 heterozygous mice. Genetics. 2007;177: 307–311. doi: 10.1534/genetics.107.075960 17890364 10.1534/genetics.107.075960 PMC2013712

[48] Yoshiki A, Moriwaki K. Mouse phenome research: implications of genetic background. ILAR J. 2006;47: 94–102. 16547366 10.1093/ilar.47.2.94

[49] Mao M, Smith RS, Alavi MV, Marchant JK, Cosma M, Libby RT, et al Strain-dependent anterior segment dysgenesis and progression to glaucoma in Col4a1 mutant mice. Invest Ophthalmol Vis Sci. 2015;56: 6823–6831. doi: 10.1167/iovs.15-17527 26567795 10.1167/iovs.15-17527 PMC4627250

[50] Behesti H, Holt JK, Sowden JC. The level of BMP4 signaling is critical for the regulation of distinct T-box gene expression domains and growth along the dorso-ventral axis of the optic cup. BMC Dev Biol. 2006;6: 62 doi: 10.1186/1471-213X-6-62 17173667 10.1186/1471-213X-6-62 PMC1764729

[51] Moreno-Miralles I, Ren R, Moser M, Hartnett ME, Patterson C. Bone morphogenetic protein endothelial cell precursor-derived regulator regulates retinal angiogenesis in vivo in a mouse model of oxygen-induced retinopathy. Arterioscler Thromb Vasc Biol. 2011;31: 2216–2222. doi: 10.1161/ATVBAHA.111.230235 21737784 10.1161/ATVBAHA.111.230235 PMC3184390

