# Supplementary figures and images for: Ciliary margin-derived BMP4 does not have a major role in ocular development
Source: PLoS One. 2018 May 8;13(5):e0197048. doi: 10.1371/journal.pone.0197048 (PMC5940228; doi:10.1371/journal.pone.0197048)

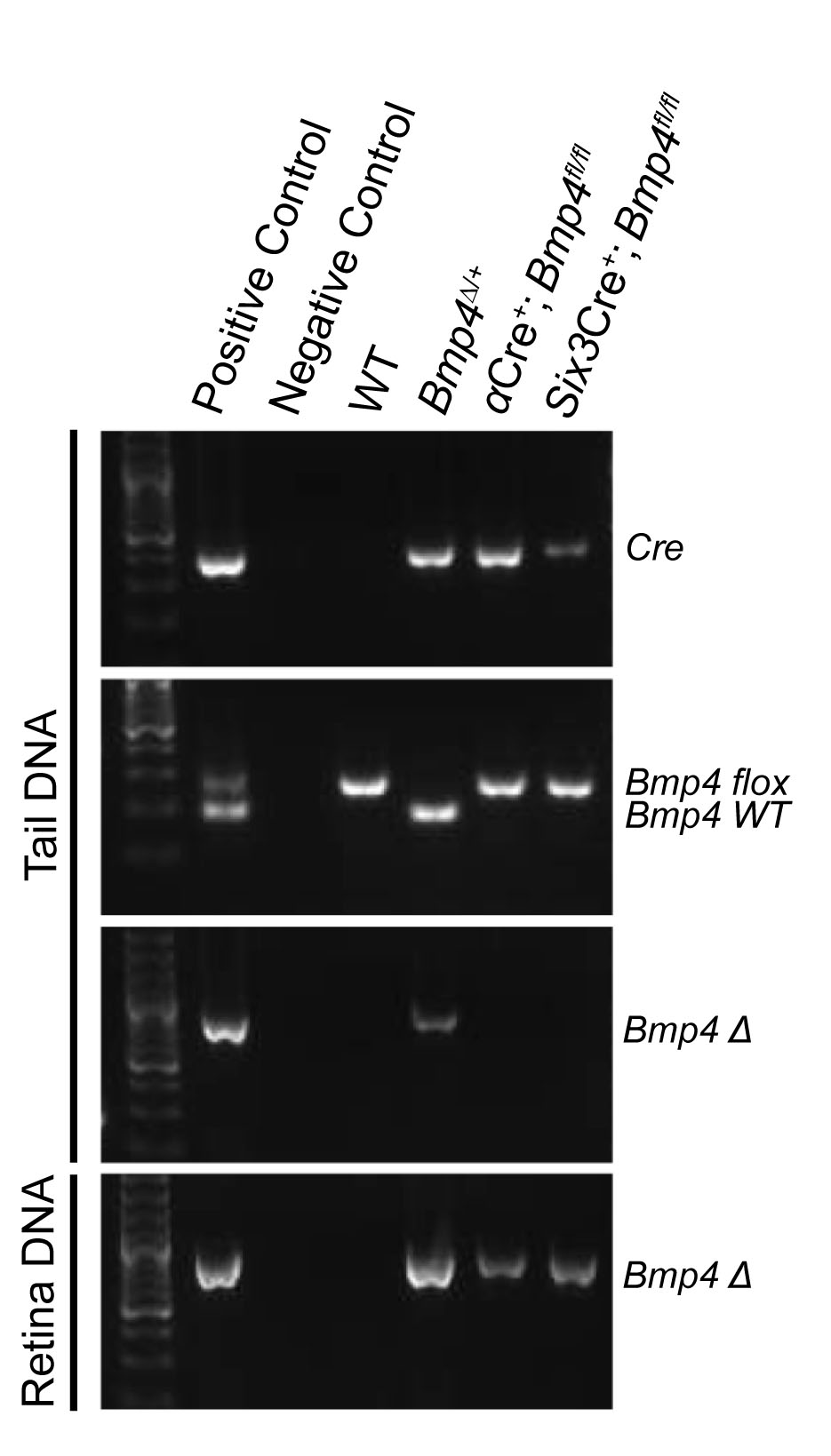

Supplement: S1 Fig — Germline deletion does not occur in conditional knockouts. Genotyping results show presence or absence of Cre, Bmp4flox, WT, and Bmp4 recombined alleles in DNA extracted from the tail and the retina of adult WT mice, αCre+; Bmp4fl/fl mice, Six3Cre+; Bmp4fl/fl mice and mice heterozygous for a null allele of Bmp4. Note the absence of the deleted band in tail DNA from αCre+; Bmp4fl/fl mice and Six3Cre+; Bmp4fl/fl mice, indicating germline recombination had not occurred. (TIF) [file pone.0197048.s001.tif]

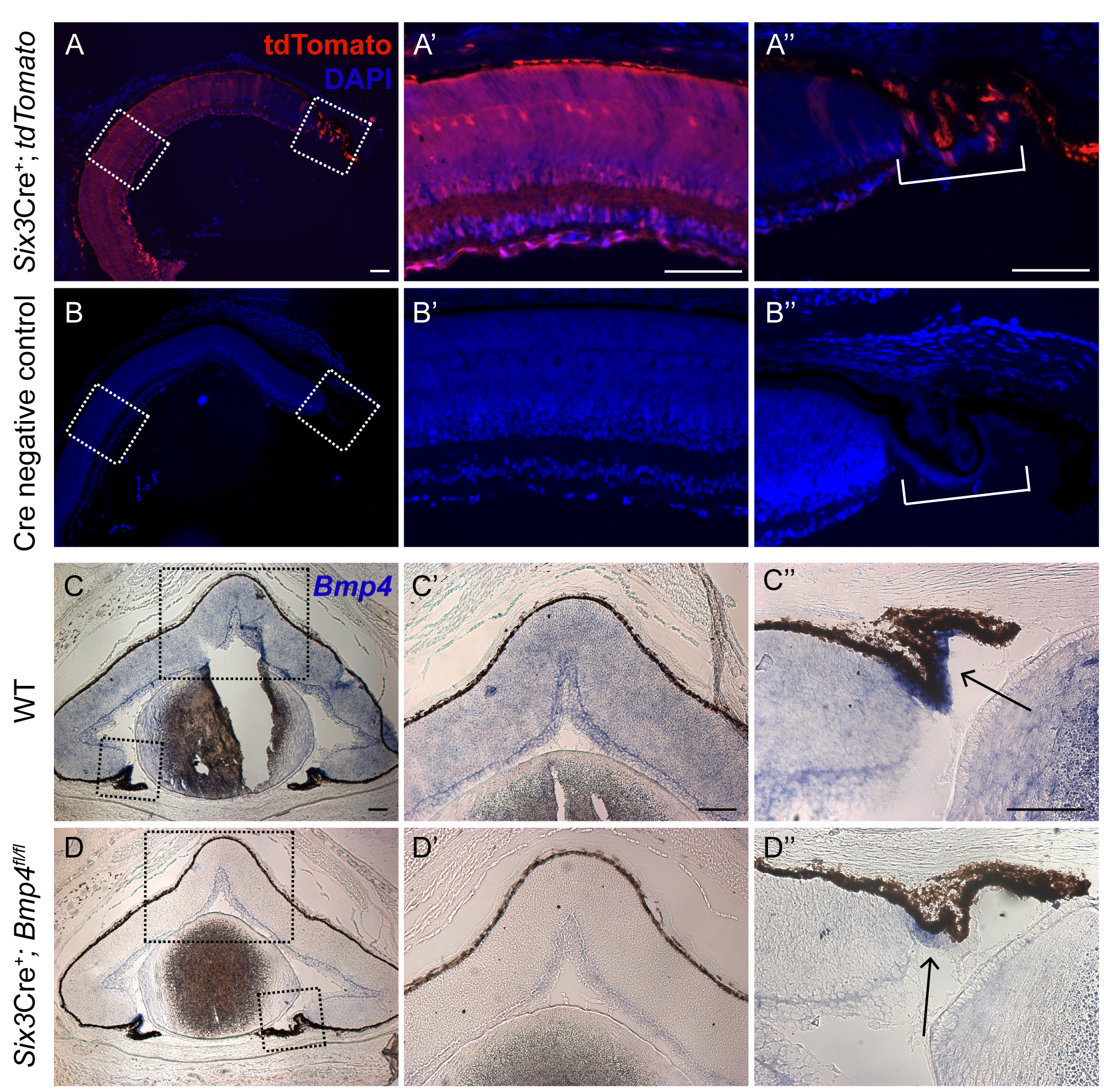

Supplement: S2 Fig — Bmp4 is efficiently removed from the central retina in Six3Cre-mediated conditional knockouts. TdTomato reporter expression driven by Six3Cre is detected throughout the central retina (A, A’) and sporadically in the peripheral retina and ciliary body (A, A”) in P3 mice. No reporter expression is seen in Cre negative controls (B-B”). Brackets denote ciliary body. (C-D”) Sections of P1 WT and Six3Cre+; Bmp4fl/fl mice were hybridized with a probe specific for Bmp4. In WT eyes, Bmp4 mRNA is present at low levels throughout the central retina (C, C’) and robustly in ciliary body (C”). Bmp4 mRNA is absent in the retina of Six3Cre+; Bmp4fl/fl mice (D, D’) and reduced in the ciliary body (D”). Arrows point to ciliary body. n = 3 per genotype per experiment. Dashed boxes in (A), (B), (C), and (D) are enlarged in panels to the right. Different sections from the same eye are shown in C-C” as well as D-D” due to sectioning/processing artifacts. Scale bars represent 100μm in all panels. (TIF) [file pone.0197048.s002.tif]
